# Response surface modeling of lead (׀׀) removal by graphene oxide-Fe_3_O_4_ nanocomposite using central composite design

**DOI:** 10.1186/s40201-016-0243-1

**Published:** 2016-01-22

**Authors:** Mohammad Khazaei, Simin Nasseri, Mohammad Reza Ganjali, Mehdi Khoobi, Ramin Nabizadeh, Amir Hossein Mahvi, Shahrokh Nazmara, Elham Gholibegloo

**Affiliations:** Department of Environmental Health Engineering, School of Public Health, Tehran University of Medical Sciences, Tehran, Iran; Center for Water Quality Research, Institute for Environmental Research, Tehran University of Medical Sciences, Tehran, Iran; Center of Excellence in Electrochemistry, Faculty of Chemistry, University of Tehran, Tehran, Iran; Biosensor Research Center, Endocrinology & Metabolism Molecular-Cellular Sciences Institute, Tehran University of Medical Sciences, Tehran, Iran; Department of Medicinal Chemistry, Faculty of Pharmacy and Pharmaceutical Sciences Research Center, Tehran University of Medical Sciences, Tehran, 14176 Iran; Center for Solid Waste Research, Institute for Environmental Research, Tehran University of Medical Sciences, Tehran, Iran; Department of Chemistry, Faculty of Science, University of Zanjan, Zanjan, Iran

**Keywords:** Graphene oxide, Adsorption, Lead (II), Optimization, Central composite design

## Abstract

**Background:**

Magnetic graphene oxide (Fe_3_O_4_@SiO_2_-GO) nanocomposite was fabricated through a facile process and its application as an excellent adsorbent for lead (II) removal was also demonstrated by applying response surface methodology (RSM).

**Methods:**

Fe_3_O_4_@SiO_2_-GO nanocomposite was synthesized and characterized properly. The effects of four independent variables, initial pH of solution (3.5–8.5), nanocomposite dosage (1–60 mg L^−1^), contact time (2–30 min), and initial lead (II) ion concentration (0.5–5 mg L^−1^) on the lead (II) removal efficiency were investigated and the process was optimized using RSM. Using central composite design (CCD), 44 experiments were carried out and the process response was modeled using a quadratic equation as function of the variables.

**Results:**

The optimum values of the variables were found to be 6.9, 30.5 mg L^−1^, 16 min, and 2.49 mg L^−1^ for pH, adsorbent dosage, contact time, and lead (II) initial concentration, respectively. The amount of adsorbed lead (II) after 16 min was recorded as high as 505.81 mg g^−1^ for 90 mg L^−1^ initial lead (II) ion concentration. The Sips isotherm was found to provide a good fit with the adsorption data (K_S_ = 256 L mg^−1^, n_S_ = 0.57, q_m_ = 598.4 mg g^−1^, and R^2^ = 0.984). The mean free energy E_ads_ was 9.901 kJ/mol which confirmed the chemisorption mechanism. The kinetic study determined an appropriate compliance of experimental data with the double exponential kinetic model (R^2^ = 0.982).

**Conclusions:**

Quadratic and reduced models were examined to correlate the variables with the removal efficiency of Fe_3_O_4_@SiO_2_-GO. According to the analysis of variance, the most influential factors were identified as pH and contact time. At the optimum condition, the adsorption yield was achieved up to nearly 100 %.

## Background

Effluents containing Lead and other toxic metals (׀׀) are increasingly discharged into the water supplies due to the expansion of industries [1]. The maximum levels lower than 15 ppb for lead (׀׀) in drinking waters has been mandated by many environmental agencies and national standard organizations [2–4]. The strict limitations on discharging effluents contained lead (׀׀) to the natural water bodies are attributed to the lead (׀׀) potential health effects on children and adults [3].

Many processes such as precipitation, membrane filtration, adsorption, and ion exchange have been applied to remove lead (׀׀) and other toxic metals from the industrial effluents [5]. Only a few methods such as using functionalized adsorbents and membrane technologies can be adopted to capture low concentrations around 1 mg L^−1^, which is commonly occurred in drinking water sources [6]. Although, adsorption processes are useful in removal low concentrations of metal ions from aqueous solutions, but there are two main limitations regarding to the use of them; 1. low adsorption capacity [7, 8], and 2. difficult separation of adsorbent from treated water after the end of adsorption process [9–11].

Graphene oxide is an emerging carbon-based nonmaterial that has revealed the promising adsorptive properties [12, 13]. Graphene oxide (GO) creates a highly stable aqueous dispersion which prepares an excellent situation for effective contacts with target contaminants without needing to vigorous mechanical mixing [14]. The GO flakes have high specific surface area ranging from 600 to 3500 m^2^ g^−1^ [15, 16]. The dispersibility property of GO is attributed to the plenty of hydrophilic functional groups on the GO flakes [15]. The GO flake surface contains different functional groups including epoxide and hydroxide, whereas, the edge of flakes are mainly contained a hedge of carboxylic groups [14].

Using magnetic agents like Fe_3_O_4_ has been considered as a way to separate the GO nanosheets from aqueous solution when the adsorption process is finished [17, 18]. Some methods employed for the adding of Fe_3_O_4_ on the GO surface are generally led to form reduced GO (rGO) [19, 20]. Because of the elimination of functional groups during the reduction process, rGO represents weak dispersity [14]. Hence, preserving the GO dispersibility in the aqueous solution as well as adding the magnetic property for separation purposes is under consideration.

Few literatures were reported applying non reduced Fe_3_O_4_/GO for the adsorption purposes [17, 21, 22]. Among them, some synthesis approaches have relied on the formation of covalent bonds between the GO sheets and Fe_3_O_4_ nanoparticles [17, 22] which has more stability than those methods based on physical attraction [21].

This research aimed to fabricate the covalent bond Fe_3_O_4_@SiO_2_-GO nanocomposite as a highly dispersible and easy separatable adsorbent for the elimination of lead (׀׀) from aqueous solution. Other purpose of the study was determining the optimal operational condition using response surface methodology (RSM) to achieve satisfactory lead (׀׀) removal. The conventional optimization method, which altered one variable at a time by keeping the other variables constant, is a time consuming and costly approach that can not consider the interactive effects between variables. RSM technique is an empirical statistical approach used to evaluate the relationship between a set of controlled experimental variables and observed results. It can be applied to optimize and identify the performance of adsorption process. Minimum experimental runs are achievable by using RSM. Applying RSM reduces the experiment runs and the reagents consumption. It also facilitates the execution of experiments necessary for the construction of the response surface.

## Methods

### Materials

Graphite powder (particle size ˂ 20 μm), tetraethyl orthosilicate (TEOS), (3-aminopropyl) triethoxysilane (APTES), n- hydroxysuccinimide (NHS) and 1- ethyl-3- (3-dimethyl aminopropyl) carbodiimide (EDC.HCl) were purchased from Sigma- Aldrich, Ltd. Co. All other chemicals such as sodium nitrate (NaNO_3_), potassium permanganate (KMnO_4_), sulfuric acid (H_2_SO_4_), hydrochloric acid (HCl), hydrogen peroxide aqueous solution (H_2_O_2_), iron chloride hexahydrate (FeCl_3_, 6 H_2_O), and iron chloride tetrahydrate (FeCl_2_, 4 H_2_O) were of reagent grade and used without further purification.

### Preparation of graphene oxide (GO)

Graphene oxide was synthesized from the graphite powder by the modified Hummers et al. method [23]. Briefly, 2.0 g of graphite powder and 2.0 g of NaNO_3_ were mixed with 92 mL of H_2_SO_4_ (98 %) in a flask and stirred in an ice bath vigorously for 0.5 h, and then 12.0 g of KMnO_4_ was added to the above solution slowly. After stirring for 0.5 h, the ice bath was removed and the solution was stirred in a water bath at 35 °C for 6 h. After that, 160 mL of the DI water was added slowly to the flask. Then, the obtained mixture was stirred at 90^0^Cfor 2 h. Afterward 400 mL of DI water was added and followed by addition of 12 mL of H_2_O_2_ (30 %), Upon which the color of mixture turned to bright yellow. The obtained suspension was washed with 1:10 HCl solution (150 mL) and DI water several times to remove metal ions [24]. The resultant dispersion was sonicated at 130 KHz for 2 h and centrifuged to obtain exfoliated graphene oxide [25].

### Preparation of Fe_3_O_4_@SiO_2_-NH_2_

The Fe_3_O_4_ magnetic nanoparticles were synthesized using a coprecipitation method [26]. For the synthesis of Fe_3_O_4_@SiO_2_- NH_2_, 1.0 g of the obtained Fe_3_O_4_MNPs was dispersed in a mixture of 40 mL ethanol and 10 mL of DI water using an ultrasonic water bath. After that, o.5 mL TEOS and 2 mL NH_3_.H_2_O (25 %) were added, and the mixture was stirred at 50 °C for 6 h. The solid product was collected by an external magnetic field, washed with ethanol and dried under vacuum. In the next step, 1 g of the obtained Fe_3_O_4_@SiO_2_ was dispersed in 25 mL dried toluene and treated with addition of 1 mL APTES [27]. The mixture was refluxed for 24 h under nitrogen atmosphere. The product was washed with ethanol and then dried to obtain Fe_3_O_4_@SiO_2_-NH_2_ [28].

### Preparation of Fe_3_O_4_@SiO_2_-GO

**Fig. 1:**
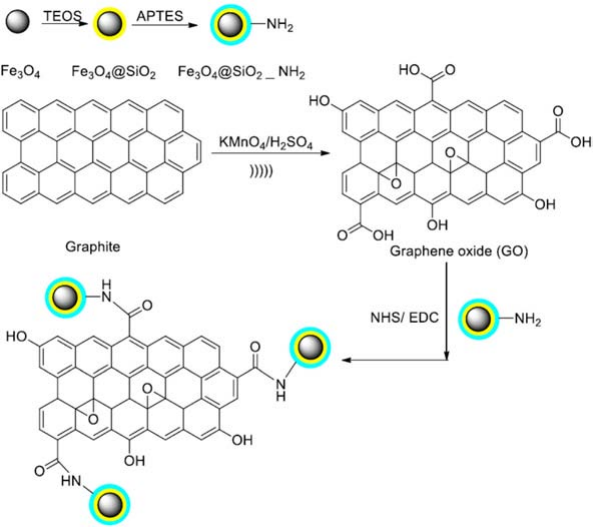
Schematic of the chemical path to the synthesis of Fe_3_O_4_@SiO_2_-GO nanocomposite. (TEOS: tetraethylorthosilicate, APTES: 3 aminopropyltriethoxysilane)

The condensation reaction between amine groups of Fe_3_O_4_@SiO_2_-NH_2_ and carboxyl groups of GO was performed [28]. Typically, 0.2 g GO was dispersed in 50 mL DI water containing of 0.1 g NHS and 0.2 g EDC.HCl by ultrasonication for 2 h. Subsequently, 0.5 g Fe_3_O_4_@SiO_2_- NH_2_was added to the above mixture and stirred for 12 h at room temperature. The solid product was collected and washed with DI water and ethanol by magnetic separation and then dried under vacuum [17, 29]. Figure 1 shows a schematic view of the synthesis process.

To confirm the stability of nanocomposite, concentration of Iron after the adsorption process was measured. As shown in Tables 2, and 3, the leaching of Iron into the aqueous solution after contact times was negligible.

### Characterization

The SEM images were taken with Hitachi- S4160 scanning microscope (Tokyo, Japan) to survey the morphological pattern and surface structural aspects of GO and Fe_3_O_4_@SiO_2_-GO nanocomposite. A Nanoscope V multimode atomic force microscope (Veeco Instruments, USA) were used to perform AFM measurements. The AFM images were taken from samples which prepared by deposition a dispersed GO/methanol solution (70 mg mL^−1^) onto a mica surface and allowing them to dry in air [25]. The images were taken under ambient condition by adjusting the instrument on the tapping mode.

### Batch adsorption experiments

Using a thermostatic shaker, batch experiments were conducted in 100 mL Erlenmeyer flasks to study the removal of lead (׀׀) on the Fe_3_O_4_@SiO_2_-GO nanocomposite. The different volumes contained known quantities of as-dispersed Fe_3_O_4_@SiO_2_-GO nanocomposite were added to 20 mL solution having predominated concentrations of Pb^2+^. All solutions underwent constant mixing at the 300 rpm for different contact times determined by the experimental design. After ending the adsorption process, the nanocomposite was eliminated from the aqueous solution by a magnet. The equation (1) was applied to determine the removal efficiency of lead (׀׀):

1 $$ \mathrm{R}\left(\%\right)=\frac{\left({C}_0-{C}_e\right)}{C_0}\times 100 $$

Where, *R (%)* is the removal efficiency, *C*_*0*_ and *C*_*t*_ are the concentrations (as mg L^−1^) of lead (׀׀) at 0 and t minutes after the contact time, respectively.

The equilibrium adsorption capacity was also obtained as equation (2):

2 $$ {\mathrm{q}}_e=\frac{\left({C}_0-{C}_e\right)}{x_{ads}}\times 1000 $$

Where, *q*_*e*_ is the equilibrium capacity (mg g^−1^), *x*_*ads*_ is the nanocomposite concentration in aqueous solution (mg L^−1^), and 1000 is converting factor (mg g^−1^).

Lead (׀׀) measurements in the aqueous solution were performed by using a Spectro Arcos ICP-optical emission spectrometer (SPECTRO Analytical Instruments, Kleve, Germany) based on radial plasma observation. The Spectro Arcos has a Paschen–Runge mount which equipped with 32 linear CCD detectors. The CCD detectors supply the ability of simultaneous monitoring of line intensities at wavelengths between 130 and 770 nm.

Isotherm and kinetic constants were obtained using the Solver “add-in” with Microsoft Excel spreadsheet program [30] according to the nonlinear forms of the equations.

### Experimental design

Central composite design (CCD) was used to investigate the lead (׀׀) removal. The RSM was employed to evaluate the combined effects of pH (*X*_*1*_), GO-Fe_3_O_4_ dose (*X*_*2*_), contact time (*X*_*3*_), and initial lead (׀׀) concentration (*X*_*4*_) on the adsorption process. The experimental conditions of independent variables, which were derived from CCD, are summarized in Table 1. The lead (׀׀) removal efficiency (*Y*) were served as output responses. Applying two blocks, one cube block and one star block, total 44 experiments were carried out, consisting of 20 center points, 2^4^ = 16 design point, and 2 × 4 = 8 axial points.

**Table 1:** The original and coded levels of independent variables

Original factors	Coded levels				
	-α	−1	0	+1	+α
pH-X_1_	3.5	4.75	6	7.25	8.5
Fe_3_O_4_@SiO_2_-GO -X_2_ (mg/L)	1	15.75	30.5	46.25	60
Time-X_3_ (min)	2	9	16	23	30
Initial Pb^2+^ Concentration-X_4_ (ppm)	0.5	1.625	2.75	3.87	5

The details of 44 experiments are presented in Table 2. The chosen independent factors applied in the study were coded based on Eq. (3):

**Table 2:** Observed and predicted values for the quadratic model (T = 298 K)^a^

Run no.	Observed values (%)	Predicted values (%)	Residual	Run no.	Observed values	Predicted values	Residual
1	53.433	57.917	−4.484	23	98.579	100.000	−1.421
2	98.904	67.699	31.205	24	90.133	83.418	6.715
3	95.230	96.303	−1.074	25	80.601	85.100	−4.500
4	95.600	23.773	71.826	26	98.995	100.000	−1.005
5	95.317	94.748	0.570	27	99.263	95.067	4.196
6	95.375	94.747	0.628	28	94.576	94.739	−0.163
7	95.626	94.757	0.869	29	94.923	94.742	0.182
8	94.884	94.749	0.135	30	89.334	88.112	1.222
9	98.796	94.709	4.087	31	98.009	100.000	−1.991
10	92.002	94.739	−2.737	32	41.095	49.543	−8.448
11	87.971	87.427	0.544	33	93.478	94.836	−1.359
12	56.850	61.083	−4.234	34	95.341	94.742	0.599
13	98.432	66.637	31.796	35	94.931	94.798	0.133
14	99.493	91.859	7.634	36	96.083	99.816	−3.733
15	94.866	94.770	0.096	37	99.695	93.757	5.938
16	99.589	54.557	45.031	38	86.848	85.821	1.026
17	95.032	94.750	0.281	39	95.115	94.757	0.359
18	95.019	94.759	0.260	40	94.402	94.747	−0.345
19	92.438	84.665	7.773	41	94.495	98.033	−3.537
20	92.624	84.440	8.184	42	95.071	94.947	0.124
21	93.237	83.804	9.433	43	95.290	94.968	0.322
22	95.020	94.797	0.223	44	99.006	100.000	−0.994

^a^The average concentration of Iron (measured by ICP-OES) after ending contact times for 44 adsorption runs: 4.32 ± 1.01 ppb

3 $$ {x}_i=\frac{\left({X}_i-{X}_0\right)}{\varDelta X} $$

Where, *x*_*i*_ is a dimensionless coded value of the *ith* independent variable, *X*_*0*_ is the center point value of *X*_*i*_ and Δ*X* is the step change value. A quadratic (second order) model as shown in Eq. (4) was applied to approximate the interaction between the response (Y) and four independent variables:

4 $$ Y={b}_0+{\displaystyle \sum_{i=1}^k}{b}_i{X}_i+{\displaystyle \sum_{i=1}^k}{b}_{ii}{X}_i^2+{\displaystyle \sum_{i=1}^{k-1}}{\displaystyle \sum_{j=2}^k}{b}_{ij}{X}_i{X}_j+c $$

Where, Y represents the dependent variable (lead (׀׀) removal efficiency), b_0_ is a constant value, b_i_, b_ii_, and b_ij_ refer to the regression coefficient for linear, second order, and interactive effects, respectively, Xi, and Xj are the independent variables, c denotes the error of prediction.

The above mentioned CCD analysis plus to the statistical analysis, such as ANOVA, F-test, and t-test were obtained using R software (version 3.0.3: 2014-03-06).

## Results and discussion

### Characterization of GO-Fe_3_O_4_ nanocomposite

**Fig. 2:**
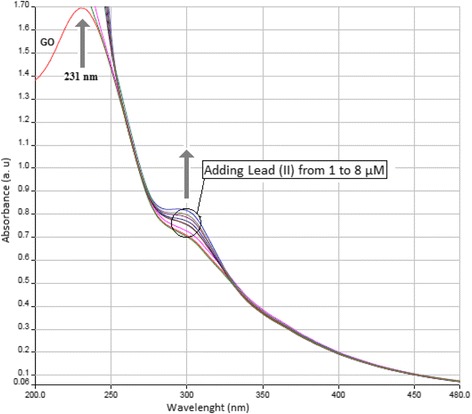
UV-visible spectra for GO dispersion in water before (*Orange line*) and after adding the different concentrations of Lead (II). Lead (II) initial concentration = 1 μM, pH = 6.8, T = 298 K

As shown in Fig. 2, a UV- visible spectrum obtained for the GO aqueous dispersion (orange line) displays a plasmon peak at 231 nm which is related to the *π* → π* transitions due to the aromatic *C* − *C* bonds. Also, a hump can be detected around 300 nm approving the *n* → π* transitions of *C* = *O* bonds [31, 32]. Gradually adding the lead (׀׀) aqueous ions into the GO dispersion resulted in producing a growing humpy pattern around 300 nm which can be attributed to the affinity between lead (׀׀) and *C* = *O* bonds relating to the carboxylic groups in the GO structure [33].

**Fig. 3:**
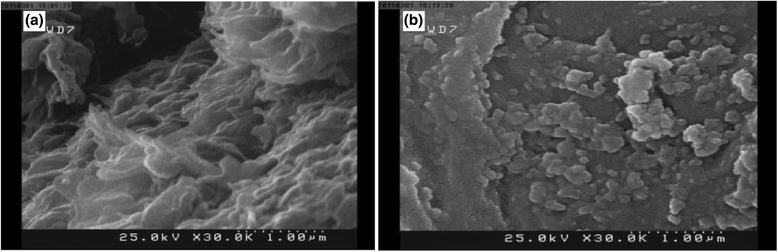
Typical FE-SEM of GO (**a**), and Fe_3_O_4_@SiO_2_-GO (**b**)

**Fig. 4:**
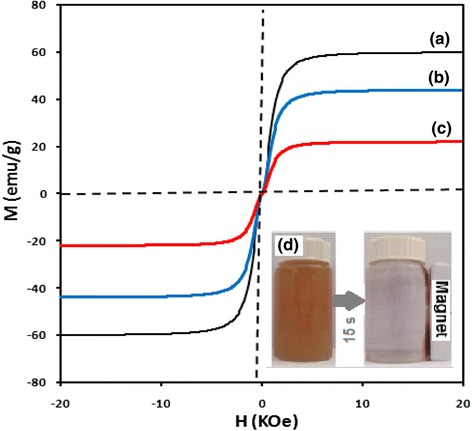
Magnetic hysteresis loops of Fe_3_O_4_ (**a**), Fe_3_O_4_@SiO_2_-NH_2_ (**b**), and Fe_3_O_4_@SiO_2_-GO (**c**). Photographic image of 1.4 mg mL^−3^ Fe_3_O_4_@SiO_2_-GO dispersions in water before (*left image*) and after (*right image*) exposing to the external magnetic field for 15 s (**d**)

Fabricated Fe_3_O_4_@SiO_2_-GO was characterized with different techniques. Figure 3 shows the field emission SEM images of GO and Fe_3_O_4_@SiO_2_-GO. Figure 3a illustrated the fluffy nature of GO layers which turned to agglomerated morphology after the formation of covalent bonds with Fe_3_O_4_@SiO_2_-NH2 and producing Fe_3_O_4_@SiO_2_-GO (Fig. 3b). The magnetic properties of the prepared nanocomposite were characterized using vibration sample magnetization (VSM). Figure 4 shows the magnetization curve patterns of Fe_3_O_4_, Fe_3_O_4_@SiO_2_-NH_2_, and Fe_3_O_4_@SiO_2_-GO. As inferred from Fig. 4, the maximum saturation magnetizations of Fe_3_O_4_, Fe_3_O_4_@SiO_2_-NH_2_, and Fe_3_O_4_@SiO_2_-GO were 60.2, 43.8, and 22.3 emu g^−1^, respectively.

The VSM curves (Fig. 4) shows that, the magnetic power of Fe_3_O_4_ nanoparticles were dropped into the one third of original state which was due to both the SiO_2_-NH_2_ coverage and the GO covalent bonds. But, the remaining 22.3 emu g ^−1^ of saturation magnetization can still be considered as a powerful magnetic field to separate the nanocomposite from the aqueous solution, as shown in the Fig. 4d. Also, the coercivity and remanence were not observed after removing the magnetic field. From Fig. 4d, the yellow brown color of the GO dispersion revealed that the oxygenation of the graphene nanosheets has been effectively occurred during the synthesis [16, 34]. After 3 months from the GO preparation, there is no any visible sign of sedimentation which shows long-term dispersibility of GO in water.

**Fig. 5:**
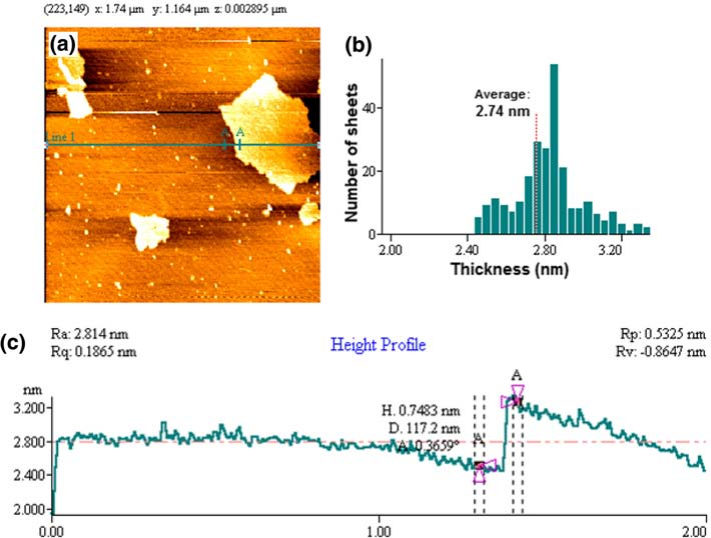
Tapered mode AFM topography scan. Exfoliated graphene oxide deposited on a freshly cleaved mica surface (**a**). Histogram of platelet thicknesses from images of 210 platelets (the mean thickness is 2.74 nm) (**b**). Height profile through the green line (*Line 1*) presented in (**a**). Cross-section A-A through the sheet shown in (**a**) exhibiting a height minimum of 0.748 nm (**c**)

The AFM image of GO nanosheets is illustrated in Fig. 5a. Also, Fig. 5b shows the distribution of GO obtaining 210 nanosheets found in a certain area of the mica surface which confirms preparing a well distributed dispersion. As shown in Fig. 5b, the average thickness measured was 2.74 nm revealed producing few layered (1 and 2 layers) GO [35].

As shown in Fig. 5c, the thickness of a random GO sheet measured using the height profile (Line 1) in the AFM image, is about 0.75 nm. This sub-nanometer thickness confirms producing the GO monolayer [14].

**Fig. 6:**
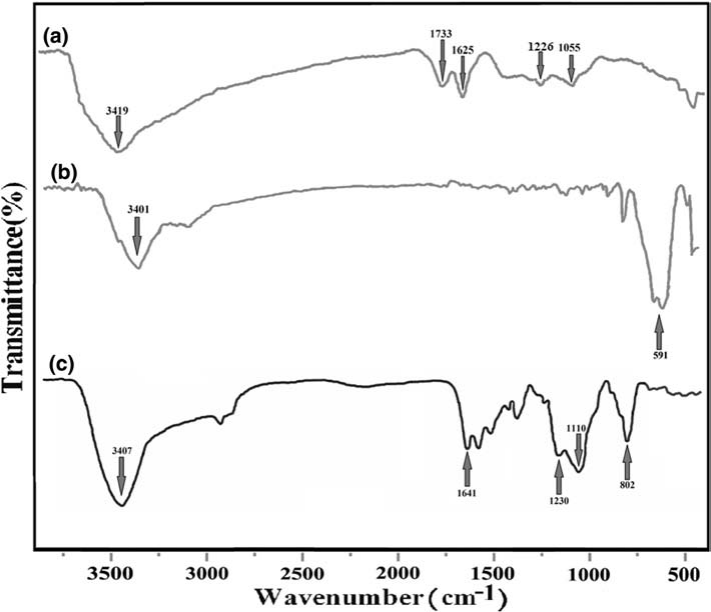
FT-IR spectra of GO (**a**), Fe_3_O_4_ (**b**)_,_ and Fe_3_O_4_@SiO_2_-GO (**c**)

Figure 6 shows the FT-IR spectra obtained for GO, Fe_3_O_4,_ and Fe_3_O_4_@SiO_2_-GO materials. Figure 6a depicts the characteristic features illustrated in the FT-IR spectrum for GO which are contained the adsorption bands attributed to the C-O stretching at 1055 cm^−1^, the C–OH stretching at 1226 cm^−1^, and the C-O carbonyl stretching at 1733 cm^1^ [36–38]. Furthermore, the O-H hydroxide stretching vibrations appear at 3419 cm^−1^. Also, the adsorbed water molecules stretching at 1621 cm^−1^, although it may feature due to the skeletal vibrations of un-oxidized graphitic remnants [39, 40].

As can be seen from Fig. 6b, the spectrum of the Fe_3_O_4_ shows the Fe-O stretching vibration at 591 cm^−1^, and an intense OH band around 3400 cm^−1^. The OH band is attributed to the stretching vibrations of Fe-OH groups attached on the Fe_3_O_4_ surface and also can be assigned to the remaining water that was not eliminated from the surface of the Fe_3_O_4_ nanoparticles [41].

As depicted in Fig. 6c, the peak at 3401 cm^−1^ of Fig. 5c attributed to the –NH_2_ vibration. Comparing Fig. 6c with Fig. 6a, the peak at 1733 cm^−1^ was almost disappeared, and a new broad peak was emerged at 1641 cm-1 corresponding to C = O characteristic stretching band of the amide group. The stretching band of the amide C–N peak appears at 1230 cm^−1^ [42]. Meanwhile, as shown in Fig. 6c, the peaks at 802 and 1110 cm^−1^ were obviously observed due to the Si–O vibrations. From these findings, it is recommended that APTES functionalized Fe_3_O_4_ was covalently bonded to GO through the amide linkage [22].

### Response surface methodology model analysis

Predicted values for lead (׀׀) removal efficiencies (%) applying quadratic model and reduced quadratic model were represented in Tables 2 and 3, respectively. The statistical significance of models was depicted in Tables 4 and 5 which represent the analysis of variance (ANOVA) for the quadratic model and reduced quadratic model, respectively.

**Table 3:** Observed and predicted values for the reduced cubic model (T = 298 K)^a^

Run no.	Observed values (%)	Predicted values (%)	Residual	Run no.	Observed values	Predicted values	Residual
1	53.433	59.426	−5.994	23	98.579	100.000	−1.421
2	98.904	68.393	30.511	24	90.133	83.437	6.696
3	95.230	96.805	−1.575	25	80.601	86.087	−5.486
4	95.600	25.705	69.894	26	98.995	100.000	−1.005
5	95.317	94.994	0.323	27	99.263	95.744	3.519
6	95.375	94.994	0.381	28	94.576	94.994	−0.418
7	95.626	94.994	0.632	29	94.923	94.994	−0.070
8	94.884	94.994	−0.110	30	89.334	89.160	0.175
9	98.796	93.827	4.969	31	98.009	94.994	3.015
10	92.002	94.994	−2.992	32	41.095	50.054	−8.959
11	87.971	86.087	1.884	33	93.478	94.994	−1.516
12	56.850	59.426	−2.577	34	95.341	94.994	0.347
13	98.432	66.406	32.026	35	94.931	94.994	−0.063
14	99.493	93.430	6.063	36	96.083	100.000	−3.917
15	94.866	94.994	−0.128	37	99.695	93.715	5.980
16	99.589	56.162	43.426	38	86.848	88.655	−1.807
17	95.032	94.994	0.038	39	95.115	94.994	0.122
18	95.019	94.994	0.025	40	94.402	94.994	−0.591
19	92.438	84.254	8.184	41	94.495	100.000	−5.505
20	92.624	84.254	8.370	42	95.071	94.994	0.077
21	93.237	83.437	9.800	43	95.290	94.994	0.296
22	95.020	94.994	0.026	44	99.006	94.994	4.012

^a^The average concentration of Iron (measured by ICP-OES) after ending contact times for 44 adsorption runs: 2.44 ± 0.12 ppb

**Table 4:** Analysis of variance (ANOVA) for the quadratic model

Model formula in rsm (X_1_,X_2_,X_3_,X_4_)	DF	Sum of squares	Mean square	F-value	Probability (P)
First-order response	4	3307.4	826.85	31.7283	<0.0001
Two-way interactions	6	999.2	166.53	6.3903	0.0002
Pure quadratic response	4	1173.2	293.30	11.2546	<0.0001
Residuals	29	755.7	26.06	-	-
Lack of fit	28	750.5	26.80	5.1449	

Multiple R^2^ = 0.879; Adjusted R^2^ = 0.820; Lack of fit: 0.337

**Table 5:** Analysis of variance (ANOVA) for the reduced quadratic model

Model term	DF	Sum of squares	Mean square	F-value	Probability (P)
pH	1	2859.39	2859.39	120.547	<0.0001
Adsorbent	1	204.08	204.08	8.6037	0.0058
Time	1	241.06	241.06	10.162	<0.0003
pH^2^	1	1165.24	1165.24	49.124	<0.0001
pH × Adsorbent	1	200.98	200.98	8.4728	<0.01
pH × Time	1	219.01	219.01	9.2330	<0.005
Adsorbent × Time	1	491.94	491.94	20.739	<0.0001
Residuals	36	853.93	23.72	-	-

Multiple R^2^ = 0.863; Adjusted R^2^ = 0.836; Lack of fit: 0.355

The reduced quadratic model was applied by omitting the variables assigned to the P-values more than 0.05 in the quadratic model [43].

The values of the determination coefficient (multiple R^2^) shown in Tables 4 and 5 indicated that 87.9 and 86.3 % of the variability in the response could be explained by the quadratic and reduced quadratic models, respectively.

If there have been various terms in the model and also the sample size has not been very large, the adjusted correlation coefficient (adjusted R^2^) may represent values considerably smaller than the multiple correlation coefficients (Multiple R^2^) [44]. In this experiment, the adjusted correlation coefficient value (adjusted R^2^ = 0.836) are also noticeable to support the high significance of the models and approves a satisfactory adjustment for the reduced quadratic model to the experimental data [45–47].

The values of the determination coefficient (multiple R^2^) shown in Tables 4 and 5 indicated that 87.9 and 86.3 % of the variability in the response could be explained by the quadratic and reduced quadratic models, respectively.

As revealed from Tables 4 and 5, the “lack of fit (LOF)” values were 0.337 and 0.355 for the quadratic and reduced quadratic models, respectively. The insignificant values of LOF (>0.05) and the significant *P*-values for both models prove that applying models is eligible to interpret the lead (׀׀) removal process and also, the reduced model is the better choice because of the higher adjusted R^2^ (0.836) and the higher value obtained for LOF (0.355) [43, 46].

Table 6 represents the regression analysis obtained from the reduced quadratic model. As shown, the significant of each coefficient was determined by *P*-value. Also, Values of “Prob > |t|” less than 0.05 in Table 6 indicate that all the model terms are significant. The bigger amounts of the t-values beside the smaller amounts of P-values show the more significant of the corresponding coefficients [46]. Based on the t- and *P* –value results, pH and time can be considered as the substantial effective factors on the lead (׀׀) adsorption. The effect of each model term, also, can be observed from the coefficient estimate values represented in Table 6.

**Table 6:** Regression analysis for the reduced quadratic model

Model term	Coefficient estimate	Std. error	t value	Pr (>|t|)	*P*-value
Intercept	−205.76106	27.09	−7.59	5.5 × 10^−9^	<0.001
pH	65.73077	7.051	9.32	3.9 × 10^−11^	<0.001
Adsorbent dosage	2.21028	0.444	4.98	1.6 × 10^−5^	<0.001
Time	4.62763	0.920	5.03	1.3 × 10^−5^	<0.001
pH^2	−3.69755	0.527	−7.01	3.20 × 10^−3^	<0.001
pH × Adsorbent	−0.19222	0.066	−2.90	6.1 × 10^−9^	<0.01
pH × Time	−0.42283	0.139	−3.04	4.4 × 10^−3^	<0.01
Adsorbent × Time	−0.05370	0.012	−4.50	5.8 × 10^−5^	<0.001

**Fig. 7:**
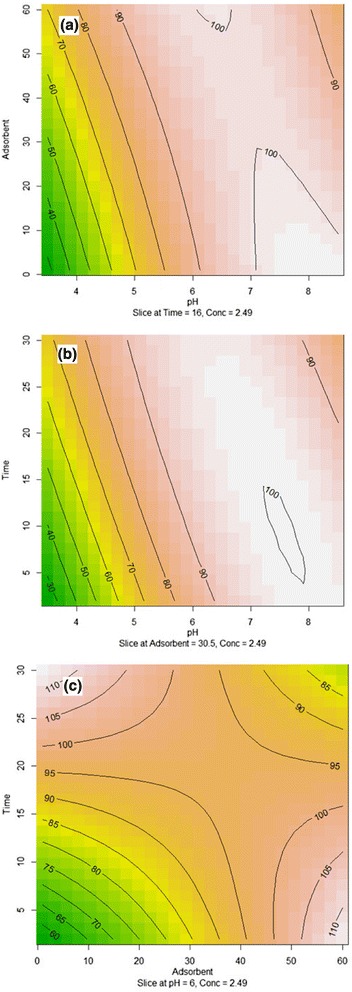
Contour plots for the effect of factors on the lead (II) removal. Adsorbent dose (mg L^−1^) and adsorption pH (**a**), contact time (min) and adsorption pH (**b**), adsorption time (min) and adsorbent dose (mg L^−1^) (**c**)

Contour plots depicted in Fig. 7 are the graphical illustrations of the regression analysis (Table 6) which represent the simultaneous effects of adsorbent-pH (a), time-pH (b), and time-adsorbent (c) on lead (׀׀) removal efficiency as the response factor. As noted above, the interaction effects of pH (X_1_) and Fe_3_O_4_@SiO_2_-GO dose (X_2_) on the lead (׀׀) removal is shown in Fig. 7a. The contact time and lead (׀׀) initial concentration were fixed constant at 16 min and 2.49 mg/L, respectively. As shown, the lead (׀׀) removal increased with increasing the Fe_3_O_4_@SiO_2_-GO dosage. The maximum lead (׀׀) removal was obtained in the range of pH from 6.5 to 8.5. In that range, the lead (׀׀) removal was independent from the adsorbent dosage. In the pH values between 3–6.5, almost a direct relationship between pH and lead (׀׀) removal was observed.

The effects of pH (X_1_) and contact time (X_3_) on lead (׀׀) removal are shown in Fig. 7b. The adsorbent dose and lead (׀׀) initial concentration were fixed constant at 30.5 mg/L and 2.49 mg/L, respectively. It is inferred from Fig. 7b that around the neutral pH, the lead (׀׀) removal process was almost completed during the contact time up to 10 min. But for pH values less than 4, after the contact time more than 30 min, the removal efficiency near 70 % was achieved.

The effects of Fe_3_O_4_@SiO_2_-GO dosage (X_2_) and contact time (X_3_) on lead (׀׀) removal can be observed in Fig. 7c. The pH and lead (׀׀) initial concentration were fixed constant at 6 and 2.49 mg/L, respectively. Figure 7b shows that, for the adsorbent doses more than 40 mg/L, regardless the contact time, the lead (׀׀) removal efficiency revealed the levels permanently beyond 95 %.

Lead (׀׀) removal showed to be very sensitive to changes in the pH both in low and high adsorbent dose. The removal capacity of Fe_3_O_4_@SiO_2_-GO nanocomposite was rapidly increased when the pH increased from 3.5 to 8.5; as it was also reported by Madadrang [15]. pH influences both the surface charges of the functional groups on the surface of graphene oxide and also the species of lead ion in the aqueous solution [48].

Increasing the protonation of functional groups on graphene oxide surface would be happen at acidic conditions and electropositivity of Fe_3_O_4_@SiO_2_-GO surface would retard the adsorption rate, and finally, the removal efficiency of lead (׀׀) can be reduced [18]. As inferred from Fig. 7a-b, when pH is less than 5, the lead (׀׀) removal efficiency was weak. However, the adsorption of lead (׀׀) was enhanced with the increasing pH from 5 to 8.5. Normally, the adsorption capacities of metal ions for most carbon based nanomaterials would increase with increases in the pH value. In this case, lead (׀׀) can be adsorbed onto the graphene oxide surface by reacting lead (׀׀) with − COOH and − OH groups [15, 49].

As shown in the contour plot exhibited in Fig. 7c, regardless the adsorbent dose, more than 75 % of lead (׀׀) adsorption was achieved during the contact time less than 10 min and the adsorption process was completely done after passing twenty minutes. These findings revealed that the fast adsorption rate of the lead (׀׀) can be attributed to the high affinity of lead (׀׀) ions to the hydroxide (−OH), epoxide (−O−) and carboxylic (−COOH) groups on the GO nanosheets [15, 50, 51].

The optimum values of pH, adsorbent dose, and contact time determined by applying the reduced quadratic model were 6.9, 30.49 mg/L, and 16.01 min, respectively. The optimum concentration of initial lead (׀׀) was not obtained from the reduced quadratic model because it was omitted from the model. But the results of quadratic model suggested 2.49 mg L^−1^ as the optimum value. Further studies such as isotherm and kinetic experiments were investigated according to the abovementioned optimum values obtained from the model.

### Adsorption isotherms

In order to investigate the adsorption equilibrium of lead (׀׀), isotherm models consisting of Langmuir (Eq. 5), Freundlich (Eq. 6), and Sips (Eq. 7) were applied. The original forms (nonlinear) of models can be expressed as follows:

5 $$ {q}_e=\frac{q_m{K}_L{C}_e}{1+{K}_L{C}_e} $$

6 $$ {q}_e={K}_f{C}_e^{1/{n}_F} $$

7 $$ {q}_e=\frac{q_m{\left(\ {K}_S{C}_e\right)}^{n_S}}{1 + {\left({K}_S{C}_e\right)}^{n_S}} $$

Where, q_e_ is the amount of lead (׀׀) adsorbed on the absorbent at equilibrium (mg g^−1^), C_e_ describes the equilibrium lead (׀׀) concentration (mg L^−1^), K_L_ is the Langmuir adsorption constant (L mg^−1^) and q_m_ denotes the maximum adsorption capacity attributeing to the complete monolayer coverage of the adsorbent (mg g^−1^). K_F_ is the Freundlich constant related to the maximum sorption capacity (mg g^−1^), also, n_F_ is the Frendlich constant related to the heterogeneity factor. K_S_ (L g^−1^) is the affinity constant and n_S_ denotes the surface heterogeneity. If n_S_ value is equal to the unity, the Sips isotherm is turned to the Langmuir isotherm, and consequently, the homogeneous adsorption can be modeled. Also, any deviation of n_S_ value from the unity (more than or less than unity) predicts the heterogeneous surface [52, 53].

The adsorption mechanism can be expressed by applying Dubinin–Radushkevich isotherm (Eq.8) which is based on the potential theory and assumes a Gaussian energy distribution.

8 $$ {q}_e={q}_m \exp \left(-{B}_{DR}{\epsilon}^2\right) $$

Where, ε is the Polanyi potential given by:

9 $$ \epsilon = RTln\left(1+\frac{1}{C_e}\right) $$

The *B*_*DR*_ constant (mol^2^/J^2^) is related to the mean free energy *E*_*ads*_ of the adsorption per molecule when it is transferred to the surface from infinity of the bulk phase.

10 $$ {E}_{ads}=\frac{1}{\sqrt{2{B}_{DR}}} $$

**Fig. 8:**
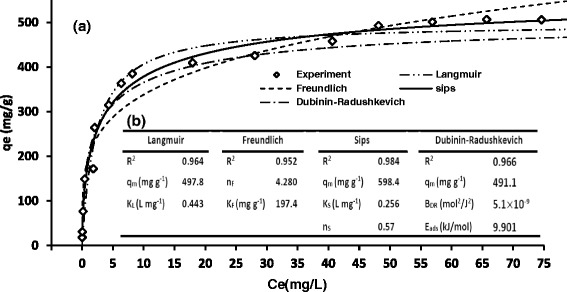
Fitting results of different isotherms for lead (II) adsorption by Fe_3_O_4_@APTES -GO (**a**), and parameters obtained for the isotherm models (**b**). *q*_m_,_exp_: 505.8, Adsorbent Dose: 30.5 mg L^−1^, pH: 6, C_0,Lead(II)_ : 0.5–90 mg L^−1^, time : 16 min

Plotting the isotherm models versus the experimental results are depicted in Fig. 8a. The parameters of isotherm models can be observed in Fig. 8b. These parameters were determined according to the nonlinear regression by plotting q_e_ versus C_e_ assisted by Solver Add-Ins MS Excel [30].

As shown in Fig. 8b, the Sips isotherm model represents the higher correlation coefficient (R^2^ = 0.984) comparing with the Langmuir (R^2^ = 0.964) and Freundlich (R^2^ = 0.952) models.

The Sips model includes three parameters and has the capability to apply for both the homogeneous and heterogeneous systems [54]. The Sips model (Eq. 5) integrates parameters from both the Langmuir and the Freundlich isotherm. The heterogeneous surface of adsorbent can be considered if the deviation of *n*_S_ value from the unity be occurred [53, 55]. However, the Sips isotherm moves toward a constant level at high concentrations whereas a pattern of Freundlich model can be observed at low concentrations [55].

According to the experimental data, the maximum adsorption uptake (q_m_) was 505.8 mg g^−1^ which indicates the adsorption capacity higher than those reported by studies applying magnetic GO as lead (׀׀) adsorbent [56] and is comparable with the studies using pristine GO [15, 49, 57]. The maximum adsorption uptake (q_m_) obtained from Sips isotherm model was found to be 598.4 mg g^−1^ which was more than the values achieved both from the Langmuir model and the experimental data (q_m.Langmuir_ = 497.8 mg g^−1^, q_m_,_exp_ = 505.8 mg g^−1^). This indicates that the Sips model overestimates the q_m_ value which can be due to the heterogeneity characteristic considered in the Sips model. As shown in Fig. 8b, the deviation of n_S_ value from the unity (n_S_ = 0.57) as well as the n_F_ value more than unity (n_F_ = 4.28) can be assigned to the crosslinking effects beside the amount of functionalities such as -COOH and -OH on the adsorbent surface (see FTIR-spectra in Fig. 6). The isotherm curves were L-shaped, which shows the high affinity of surface groups towards lead (׀׀) ions both at low and high concentrations [55]. As revealed from Fig. 8b, the mean free energy E_ads_ was 9.901 Kj/mol which seems to be the evidence of predomination the chemisorption mechanism [58].

### Adsorption kinetics

The experimental data were fitted with the original (nonlinear) forms of Lagergren-first-order (Eq. 11), pseudo-second-order (Eq. 12), and Double-exponential kinetic (Eq. 13) equations.

11 $$ {q}_t={q}_e\left(1- exp\kern0.5em \Big(-{k}_1t\right)\Big) $$

12 $$ {q}_t=\frac{K_2{q}_e^2t}{1+{q}_e{k}_2t} $$

13 $$ {q}_t={q}_e-\frac{D_1}{x_{ads}} \exp \left(-{k}_{D_1}t\right)-\frac{D_2}{x_{ads}} \exp \left(-{k}_{D_2}t\right) $$

Where, q_t_ and q_e_ are the sorption capacity (mg g^−1^) at time *t* and at the equilibrium time, respectively. k_1_ and k_2_ are the pseudo-first-order and pseudo-second-order rate constants, respectively. *D*_*1*_ and *D*_*2*_ (mg L^−1^) are the rapid and slow steps, *K*_*D1*_ and *K*_*D2*_ (min^−1^) are constants controlling the mechanism of slow and rapid phases, respectively. *x*_*ads*_ is the adsorbent dosage (g L^−1^).

**Fig. 9:**
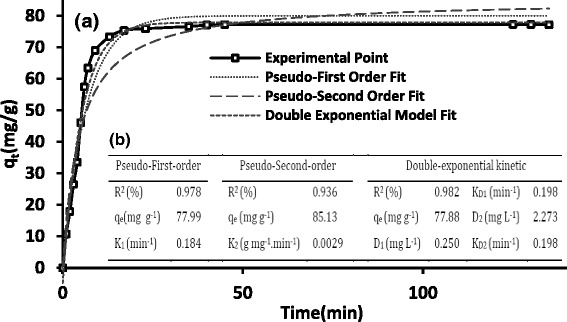
Fitting results of different kinetic models for lead (II) adsorption by Fe_3_O_4_@APTES -GO (**a**), and parameters obtained from the kinetic models (**b**). Adsorbent Dose: 30.5 mg L^−1^, pH: 6, C_0,Lead(II)_ : 2.36 mg L^−1^

Figure 9a presents the nonlinear curves attributed to the kinetic models by applying equations 11, 12, and 13. Also, the kinetic parameters of lead (׀׀) removal were illustrated in Fig. 9b.

According to the regression coefficient values of kinetic models, it was found that the double exponential kinetic model (R^2^ = 0.982) obtains a better description to predict the kinetic data of lead (׀׀) than both pseudo-first-order and pseudo-second-order models.

The values of constant parameters of double-exponential kinetic model revealed that the both external diffusion and internal diffusion have substantial effects on the lead (׀׀) sorption using Fe_3_O_4_@SiO_2_-GO nanocomposite [50].

## Conclusions

Magnetic Fe_3_O_4_@SiO_2_-GO nanocomposite was synthesized and applied to elimination the lead (׀׀) from aqueous solution. Due to the high loading capacity of GO for metal ions, Fe_3_O_4_@SiO_2_-GO revealed excellent performance in treatment the lead (׀׀) contaminated waters. The removal process was found to be quick and facile, and the lead (׀׀) adsorption process was almost completed up to 10 min contact time.

Main advantages of Fe_3_O_4_@SiO_2_-GO nanocomposite include quick separation performed by using an external magnetic field and the noticeable lead (׀׀) removal capacity (506 mg g^−1^).

A central composite design (CCD) was applied to investigate the effects of four adsorption variables, namely pH, adsorbent dose, contact time, and initial lead ion concentration on the removal efficiency of lead (׀׀).

Both the quadratic and reduced quadratic models were applied to correlate the variables to the response values. Results from the analysis of response surfaces indicated that pH, time, and the adsorbent dose were found to have significant effects on the removal efficiency of lead (׀׀). The optimization of process was performed and the experimental values were found to be agreed satisfactorily with the predicted values.

The adsorption isotherms and kinetics were also investigated. Equilibrium adsorption data had best fit by the Sips isotherm model and chemisorption mechanism was predominated. Kinetic studies indicated that the double-exponential kinetic model is the preferred model to explain the equilibrium adsorption over the time.
